# Scanning Laser Rangefinders for the Unobtrusive Monitoring of Gait Parameters in Unsupervised Settings

**DOI:** 10.3390/s18103424

**Published:** 2018-10-12

**Authors:** Sebastian Fudickar, Christian Stolle, Nils Volkening, Andreas Hein

**Affiliations:** 1 Department of Health Services Research, CvO University Oldenburg, School of Medicine and Health Science, 26111 Oldenburg, Germany; christian.stolle1@uni-oldenburg.de; 2 OFFIS e.V., 26121 Oldenburg, Germany; nils.volkening@offis.de (N.V.); andreas.hein@offis.de (A.H.)

**Keywords:** gait recognition, scanning laser rangefinders (SLR), GAITRite, cadence, velocity and stride-length

## Abstract

Since variations in common gait parameters (such as cadence, velocity and stride-length) of elderly people are a reliable indicator of functional and cognitive decline in aging and increased fall risks, such gait parameters have to be monitored continuously to enable preventive interventions as early as possible. With scanning laser rangefinders (SLR) having been shown to be suitable for standardised (frontal) gait assessments, this article introduces an unobtrusive gait monitoring (UGMO) system for lateral gait monitoring in homes for the elderly. The system has been evaluated in comparison to a GAITRite (as reference system) with 86 participants (ranging from 21 to 82 years) passing the 6-min walk test twice. Within the considered 56,351 steps within an overall 7877 walks and approximately 34 km distance travelled, it has been shown that the SLR Hokuyo UST10-LX is more sensitive than the cheaper URG-04LX version in regard to the correct (automatic) detection of lateral steps (98% compared to 77%) and walks (97% compared to 66%). Furthermore, it has been confirmed that the UGMO (with the SLR UST10-LX) can measure gait parameters such as gait velocity and stride length with sufficient sensitivity to determine age- and disease-related functional (and cognitive) decline.

## 1. Introduction

The prolongation of elderly people’s ability to remain independent in their common environments is an essential necessity to assure both a high quality of living for elderly people and well-functioning health-care systems. Thus, the early detection of functional decline and a deep understanding of elderly people’s locomotion processes are both essential aspects for the prevention of falls, which are the leading cause of fatal injury and the most common cause of non-fatal trauma-related hospital admissions among older adults causing over $50 billion total medical costs in 2015 [1] and being critical for losing the ability for independent living due to the resulting potentially severe effects on patients’ physical and mental health [2,3,4,5,6]. Thus, the reliable identification of at-risk patients at the earliest possible stage is a critical foundation to initiate appropriate preventive interventions [7] and thereby prolong functional and cognitive decline. The human gait (e.g., quantified as gait speed) has been confirmed to be a comprehensive measure and indicator for both, functional and cognitive decline [8,9]. For example, Savica et al. have investigated GAITRite measures of dementia patients and found an association between reduced gait velocity, cadence and stride length, and both global and domain-specific cognitive decline [10]. Similarly, Bridenbaugh proposed that stride speed and variability may be sensitive enough to track cognitive impairments [11].

**Figure 1:**
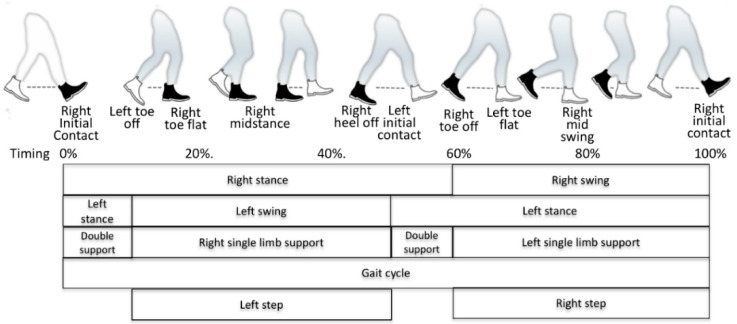
Gait cycle with the corresponding events (along with a typical ratio-timing based on occurrence within a gait cycle), the corresponding phases; black shoe represents right foot.

A gait can be characterised via the following events and parameters. As shown in Figure 1, a gait cycle or stride is defined as the phases during movement of both, the left and the right feet once. In contrast, a step considers the movement of either the left or the right foot (starting with toe off/last contact and lasting till initial contact of the same foot). Thus, a stride consists of two steps. Common parameters to characterise a gait typically consider a walk, which consists of multiple sequential steps. Common parameters (listed in Table 1) such as the cadence, velocity, stride length (L/R), L/R stance phases, L/R swing phase, L/R step length and step width are calculated from the durations and distances of the stance and swing phases and the foot positions, in accordance with the following equations. Among these parameters, the latter five parameters can be derived separately for each foot.

A study indicated a standard deviation of 7% among for normal indoor gait velocities (as shown in Table 2) ranging (in mean) between 80–91 m/min for men and 73–81 m/min for women [12,13].

In contrast, age-related variability of the gait (e.g., due to corresponding disabilities such as arthritis) was shown for gait velocity to be ranging from 14% [14] to 29% for the transition to frailty and even 51% in case of fearful fallers. This decrease of velocity has been shown to be mainly related to a decreased stride length of approximately 10% [15,16] and even 21% for transition to frailty and 41% for fearful fallers. In contrast, the cadence shows rather minor variations among these age-related phenomena.To summarise, aging in gait can be expected to manifest in smaller rather than in less steps.

However, functional decline in terms of variations in the gait initially occur during daily activities and not during phases of peak performance within clinical assessments, for the following reasons: While being conducted in rather standardised settings and thereby assuring performance comparability among elderly people, well-accepted clinical assessments are typically not applied in a preventive manner. Furthermore, elderly people’s performance within clinical assessments has been shown to be affected by the physicians’ presence. In addition, due to the incomparability of clinical walkways with rather complex home environments, elderly people’s performance within clinical tests is less representative of their common performance [17]. Consequently, clinical tests have been shown to result on average in 21% higher walking speeds and a mean 6% higher step-length than experienced in everyday living scenario tests and thereby have been shown to allow only limited insights into the elderly people’s everyday performance [17].

Consequently, with gait being a suitable early indicator of functional and cognitive decline and being specifically sensitive in unsupervised everyday living conditions, corresponding sensitive measurement technology is required [17] that supports a frequent sufficiently sensitive unsupervised technical monitoring (and assessment) of physical performance in domestic environments, as argued by Hellmers et al. [18]. In order to be accepted for continuous monitoring of the elderly people’s activities within their homes, these sensors have to preserve the participants’ privacy and have to consider users’ low technological readiness [19], while being sufficiently robust in regard to context variations.

While accurate gait analytic-systems such as the GAITRite [23] and the Vicon [24] system are commonly accepted as gold standards, they are rather unsuitable for long-term monitoring especially within domestic environments due to the associated costs and installation efforts. In regard to the unobtrusive monitoring of the gait in domestic environments, various senor types have been considered. The sensitivity of passive infrared (PIR) presence-sensors or light-barriers, due to the low granularity of the gathered information, still has to be confirmed [19,25]; and body-worn inertial measurement units (IMU) [26,27] require users’ willingness and habituality of use. The following ambient sensing techniques fulfill the aforementioned criteria much better. For fine-grained insights of the gait, scanning laser rangefinders (SLR) achieve sufficient sensitivity to characterise the gait (see Table 3). SLRs send radial horizontal laser beams and detect the distance to obstacles based on the time-of-flight that the returning beams (as being reflected by these obstacle) take until being received by the SLR. Within, the angular resolution of SLRs refers to the amount of samples taking per degree. The amount of reflected laser points determine the resolution of obstacles.

As summarized in Table 3, most systems for automated detection of gait parameters apply SLRs, followed by systems that use red-green-blue (RGB) (D) cameras. Most approaches use a Kalman filter in combination with a background subtraction procedure and hidden Markov models for classification. In most cases, the SLR are aligned parallel to the walking direction of the subject and at a height of 20–40 cm, which corresponds approximately to the height of the knee or shin. No publication was found that placed the SLR orthogonally to the walking direction. In most publications, only subsets of the relevant gait parameters are described or evaluated, and the given parameters are usually also very specifically adapted to the respective scenario, which makes comparison difficult in many places. The number of subjects in most studies is in the single-digit range, rarely in the low to medium double-digit range. The age of the test persons also varies greatly from young adults (22–30 years) to seniors (67–92 years).

However, to the authors’ best knowledge, so far only the application with a frontal placement of the SLR towards the walking patients has been investigated. While the frontal placement is suitable in a supervised clinical setting, it has limited practicability within everyday home scenarios, where movements have to be characterisable from the side. In addition, the sensitivity of both SLR types, the Hokuyo URG-04LX (herein referred to as SLR-04) and the Hokuyo UST-10LX (herein referred to as SLR-10) in regard to sensing gait parameters have yet to be compared.

Thus, this article introduces an SLR-based autonomous in-home sensor-system for unobtrusive long-term and privacy-preserving in gait monitoring that overcomes the requirement of frontal sensor placements. The system’s sensitivity and both SLRs’ sensitivity is evaluated in comparison to GAITRite measures (as reference system) with 86 subjects.

## 2. Materials and Methods

In this Section, we initially introduce unobtrusive gait monitoring (UGMO), which is followed by the description of the study design and the evaluation methodology.

### 2.1. Unobtrusive Gait Monitoring (UGMO)

**Figure 2:**
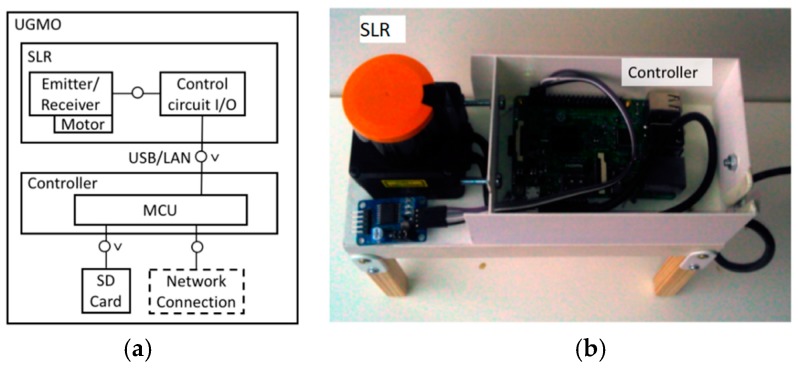
The resulting unobtrusive gait monitoring (UGMO) system: (**a**) the system components, (**b**) the device.

The developed UGMO system (shown in Figure 2) has been designed for unsupervised sensitive monitoring (and assessment) of common gait-parameters within domestic environments. In order to affect the proband as little as possible, the device has a reasonably small size, and does not require any user interaction. Furthermore, UGMO can automatically detect time-series with gait activity and will only record these movements. The UGMO system consists of a measuring platform and a software based signal-processing chain, as described subsequently.

#### 2.1.1. UGMO Hardware

The UGMO hardware combines a Hokuyo SLR with an ambient light sensor and a processing unit (based on a Raspberry Pi 3) within a handy form factor (L × H × W: 20 cm × 20 cm × 8 cm). By utilizing these sensors, the users’ privacy is assured since only gait-related distance measures of feet and intensity of ambient lights are monitored.

The SLR is placed at a height of approximately 15 cm above ground, since being suitable for gait measurements. Two popular versions of the Hokuyo sensors—the SLR-04 and the SLR-10 have been evaluated for comparison. While the cheaper SLR-04 has been successfully applied for gait recognition [28,29,30], the SLR-10 could be expected to achieve higher sensitivity due to its four-fold scanning rate and coverage of an increased measuring area (as summarized in Table 4)—which becomes especially relevant in the case of scanning the gait of bypassing persons. However, it comes with a 40% increased price and thus might be considered only if achieving significantly higher results.

Intended as a monitoring device, UGMO can either operate as standalone (by recording to a memory card) or can transmit recordings directly to the Internet. The UGMO’s data recordings support sufficiently long recording durations: with each measure holding approx. 13 KB (resulting in 52 KB/s for SLR04 and 130 KB/s for the SLR10) and each walk (over a distance of approx. 5 m) approx. 3 MB, the UGMO could record approx. 355 days on a 32 GB memory card when assuming 30 walks per day and compression adding further power of 10, the data size is unproblematic. UGMOs power connection consists of a 5V DC, 700–1000 mA power supply for the Raspberry Pi 3 and a power supply for the SLR (see Table 4). With UGMO’s hardware design being straightforward the following description focuses on the algorithmic approaches.

#### 2.1.2. Signal-Processing

**Figure 3:**
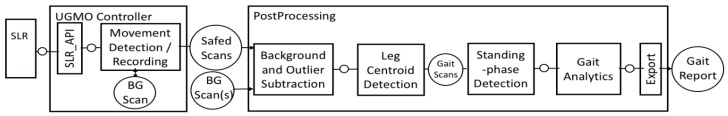
UGMO’s signal processing workflow; ranged laser scanner; BG scan: background scan; rectangles indicate processing steps and circles indicate data storages.

UGMO integrates the following signal-processing workflow (shown in Figure 3).

The software has been developed via Python 3.

The **movement detection/recording** module handles the communication with the SLR, detects when a walk has been performed in front on the UGMO and records the SLR data of movement sequences. Therefore, the module initially (and regularly) collects a background scan (BG scan) against which subsequent scans are substituted. Movement is detected if scans significantly differ from the background. In these cases, the scans are recorded for later processing or could be transferred to a server for direct analysis via an available network connection. It has two parameters (the sensitivity parameter and delay parameter) to calibrate the accuracy of the movement-detection: The sensitivity parameter defines how many measured points (see angular resolution in Table 4 for each SLR) have to be different from the background laser scan. For the SLR-10 the sensitivity parameter has been set to 3 and for the SLR-04 4, respectively. The delay parameter describes how many consecutive laser scans have to pass the sensitivity (3 for SLR-04 and 27 for SLR-10) in order to start (and end) a walk. These settings have been chosen experimentally.

**Figure 4:**
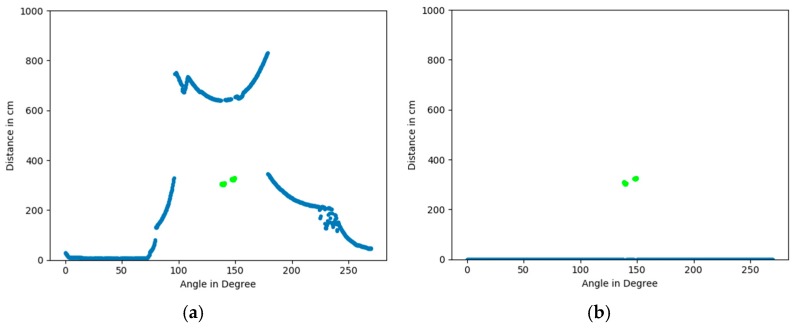
Examplary results of the background and outlier subtraction: (**a**) original image: steps (in green) and background (in blue) (**b**) results of background and outlier subtraction.

**Background and outlier subtraction:** for points that differ significantly from the background image, the presence of a movement is assumed and thus these points are further considered. Thus, the stored background image is subtracted from the current recorded (scan-) image by the following algorithm: angular points are compared among both images and if differentiating by less than a threshold (of 20 cm for the SLR-10 and 3 cm for the SLR-04), the resulting angular point-specific distant-measure is excluded from further investigations (by setting it to 0). An examplary resulting scan is shown in Figure 4b.

Next, in order to exclude temporal noise from the resulting subtracted image, only the remaining points that contain min. (5 for SLR-10, 2 for SLR-04) neighbouring points (that are not 0) are further on considered. Also, the neighboring points have to have a value difference of less than (10 cm SLR-10, 3 cm SLR-04).

**Leg centroid detection:** In order to detect legs in the remaining distance points per scan, the k-nearest neighbour (knn) clustering with an intended cluster amount of 2 is applied. Subsequently, the cluster centers are calculated from the corresponding points per cluster. These two cluster centers represent the desired ankle (talus and tibia) positions of the distant and the frontal leg. Since considering the detected leg positions of all scans, the brief loss of the back leg is unproblematic and the algorithm is as well suited to cover the particular case of covert legs, where the distant leg (from sensor point of view) is briefly covert by the frontal leg (for a few milliseconds during the swing phase of the frontal leg) meanwhile no points are collected for the distant leg. For the leg centroid detection, SLR-specific parameter-settings have not to be differentiated.

**Figure 5:**
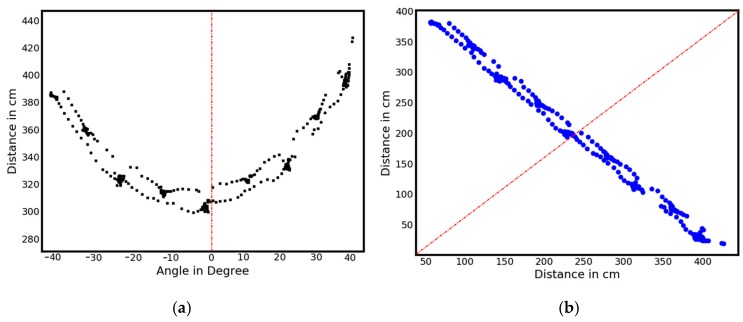
(**a**) Summary of all leg positions into a single image (**b**) after switch of coordinate system (Figure 5b: the SLR is placed at coordinate 0/0 with the center point of view facing along the f(x) = x line).

**Standing phase detection:** In order to identify the standing phases (temporal and spatial), the ankle-positions within all scans of a detected walk are condensed into a single 2D image (see Figure 5a). The previously detected ankle positions are subsequently transformed from angular- and distance-encoded vectors to concrete 2D positions via the following trigonometric functions (where α is the angle in degrees for the current SLR sample as shown in Figure 5a):

x = distance × cos (α)

y = distance × sin (α)

The resulting converted (geographically correct) visualization is shown in Figure 5b.

**Figure 6:**
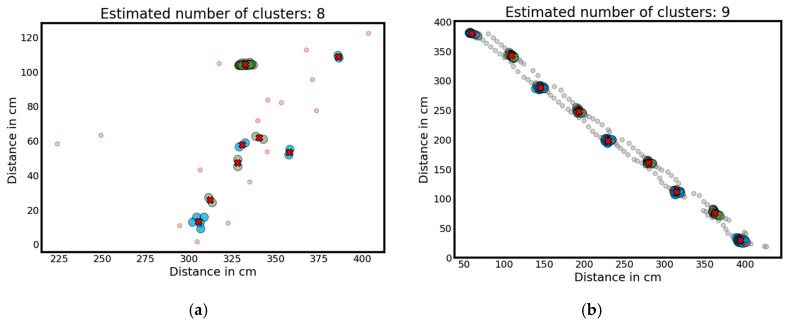
The results of the standing phase detection for an examplistical (better) identical walk representing the identified standing phases based on the DBSCAN clustering (with standing phases marked in color, general detected ankles as black dots, and corresponding standing positions marked via a red cross). (**a**) For the erroneous SLR-04 sensor and (**b**) for the SLR-10 sensor.

Since in standing positions the ankles are positioned at the same coordinates for multiple (consecutive) scans, the corresponding regions can be calculated correspondingly via these concentrations (as shown for example in Figure 6). In order to identify these concentration points, the clustering algorithm DBSCAN has been used with the following parameter settings: Its maximal distance between intra cluster-points being generally set to 50 mm and the minimal number of points per cluster being set to 2 for the SLR-04 and to 7 for the SLR-10. Exemplary results of this standing phase detection are shown in Figure 6a for the SLR-04 and Figure 6b for the SLR-10 and indicate the higher amount of ankle measures to be considered for the SLR-10 in comparison to the SLR-04.

The centers of the identified clusters are calculated as the median of the timestamps of all ankle measures per cluster. Next to the median timestamp, the corresponding x and y coordinates of this ankle measure and the cluster’s starting and ending time (representing first and last contact time) are considered of the are used for the static phase (see Figure 6). The use of the median was preferred over the first and last contact time, since it is expected to achieve more robust results.

The identified coordinates and timestamps are afterwards used within the gait analysis to extract the corresponding gait parameters.

In order to derive the common gait parameters velocity, cadence, walking distance, stance time, swing time and stride length, the contact times per standing phase (representing steps) and the associated step positions are further analysed within the **Gait Analytics** processing step. The analysis has been made using both, either the ‘First Contact Time’ or the ‘Average Contact Time’ to identify the sensitivity of both parameters. The steps for both parameters have then been matched to the GAITRite Analysis with a maximum time difference ‘timedelta’.

While the calculation of the gait parameters itself is straightforward, the following three further pre-processing steps have to be conducted.

Initially, the steps are ordered ascendingly according to the time of recording. Consecutively, the orientation of the steps (left or right leg) is calculated for the initially step by detecting whether the walk is orientated from left to right or right to left and then whether the following step has an increased or decreased distance to the SLR. The orientations of subsequent steps are then altered.

**Figure 7:**
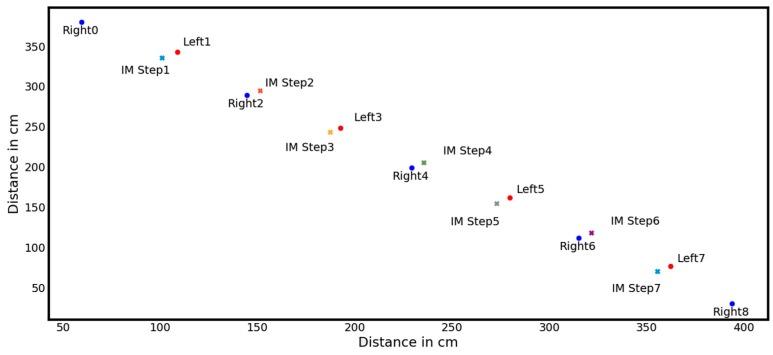
Calculation of steps as part of gait analysis (IM Steps representing calculated intermediate steps).

Furthermore, each intermediate step (IM Steps, see Figure 7), being at the midpoint of one stride and orthogonally to the position of the standing leg (as shown in Figure 7), is calculated since being required for the calculation of the single-step parameter. Intermediate steps are calculated via 3 subsequent standing phases as follows. Among the first and the third standing phases (both of this leg) a line is assumed and the step position is assumed to be the junction, where an orthogonal line to these base line passes through the position of the second standing phase (of the other leg).

In the subsequent **Calculation of Gait Parameters**, the gait parameters are then calculated as described in Section 1 Table 2 based on the extracted spatio-temporal data (x,y coordinates and timestamps) of the detected steps.

The **export** format is based on the one of the GAITRite csv export (a tab-separated ASCII export file holding the field per step as summarized in Annex 1), with the difference of it being more easily readable for a human (and the common step count encoding of GAITRite has been excluded). Complete walks are saved in one row, with individual steps being saved in lists, instead of every single step having one row. The current export contains all described gait-parameters, namely:

- Per walk: subject ID, timestamp, distance, cadence, speed (speed and GAITRite’s parameter of velocity are used synonymously);
- Per step: first contact time, last contact time, left/right stride length (cm), left/right stance phases (s), left/right swing phases.

### 2.2. Study Design

**Figure 8:**
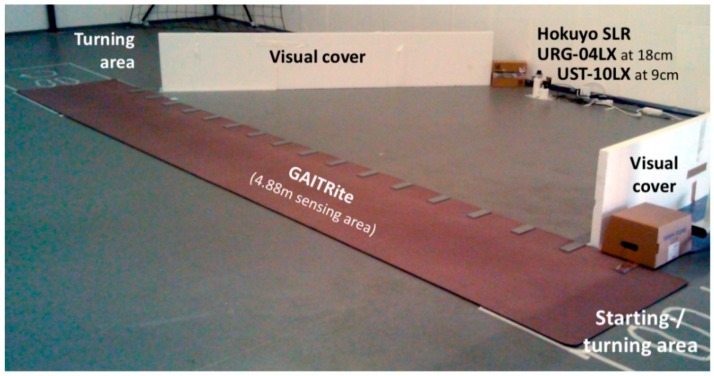
The measurement setup: Including the GAITRite and both SLRs with a visual shielding to limit their viewing angle on GAITRite’s active sensing area and exclude other walks. Participants passed the GAITRite with turning in the marked turning areas 2 times for 6 min at various paces.

In order to evaluate UGMO’s sensitivity regarding the detection of walks, steps and the corresponding gait parameters, the following study has been conducted: UGMO’s sensitivity was compared to a 6 m GAITRite (with an active sensing area of 4.88 m) with GAITRite SW Version 4.8.7, acting as reference. To clarify the suitability of both SLRs, both have been used in parallel and have been placed on top of each other (the SLR-10 at a height of 9 cm and the SLR-04 at 18 cm, respectively) facing the GAITRite walkway at a distance of 3.6 m to the GAITRite center. The brief height alteration between both SLRs is unproblematic in regard to the systems’ sensitivity, since both positions are centered around the ankle, which assures reasonable low variability regarding the measurement distances. Thereby, it is assured that all 3 sensors (the GAITRite, the SLR-04 and the SLR-10) record the same gaits. In order to achieve direct comparability among the systems, the SLR wider measurement angel (see Table 4) was restricted onto the GAITRite sensing area via visual covers as shown in Figure 8. To assure clock synchronization, all three devices were connected to the same measurement computer. The precision of the timestamps was recorded with 1000 Hz by all systems.

Within the study, the recording software was deployed on a standard PC (Windows 7 64bit 4 GB Ram, Python 3 and the GAITRite software).

Within this setting, subjects were requested to walk continuously over the GAITRite to and fro for 6 min. In order to cover a wider spectrum of walking speeds, they were requested to pass this 6 min walk test twice—once at comfortable and once at quick pace. Since the systems’ sensitivity is based on the number of scans per step, slower paces cause a higher number of scans per step than faster ones. Thus, a further investigation of slow paces was undertaken in order to overcome exhaustion of the participants as a result of the additional efforts.

We provided verbal, as well as written information for all potential participants and checked for inclusion and exclusion criteria (ability to walk with socks and without walkers and being able to pass the timed up and go (TUG) test within 10 s). The study received ethical approval by the ethical committee of the University of Oldenburg approval code number Drs. 33/2016.

### 2.3. Methodology

In order to study UGMO’s sensitivity, the measurements of the SLRs and the GAITRite have been exported as csv files (covering all foot positions per subject in millisecond accuracy). Per subject, a list of all walks measured by GAITRite, SLR-04 and SLR-10 is extracted from the respective export files. When comparing the sensitivity of the automatic gait detection (separately per SLR), the first and last step (each as first contact time) from each walk were then extracted and compared to the GAITRite steps with a synchronization overlap time-delta. For this synchronization overlap, 3 s were determined initially as suitable when considering the amount of correctly associated steps among each UGMO version and the GAITRite (see Table 5).

If the steps of the GAITRite and SLR were within this time delta, the walk was treated as a correctly detected walk. For the evaluation of the detection accuracy of individual steps, each single step (of the sorted SLR step list) was compared and treated as correct if it had a matching counterpart within the sorted GAITRite list. This was the only step, in which the results of the different sensors were synchronized for evaluation purposes. Even though the clocks were only synchronised per subjects, the clock-drift remained uncritical, since every 5 m walk was synchronised separately among all systems. In addition, the gait parameters were calculated via UGMO’s signal-processing chain per correctly detected walks. Afterwards, the parameters were averaged per walk separately for UGMO and GAITRite measures. In order to clarify the influence of gait speed on the systems sensitivity, the walks were separated into two groups according to the walking-past blocks: normal and fast walking speeds. Subsequently, the results per group were evaluated via Pearson correlation coefficient and the calculation of errors between both systems.

## 3. Results

### 3.1. Descriptive Statistics

Within the described evaluation setup, recordings of the gait of 92 subjects were recorded for evaluation purposes.

Among the recordings, six subjects were excluded due to the following errors: For two subjects the same ID has been used. Two additional subjects have not correctly executed the protocol, but have turned around already after 3 m instead of passing the full distance. For two additional subjects, no SLR measures were recorded during assessment. After initial exclusion of these six subjects the descriptive statistics (shown in Table 6) applies for the remaining 86 subjects.

Within the study overall 56,351 steps within an overall 7877 walks were recorded within approx. 8 h.

### 3.2. Influence of Scanning Laser Rangefinder (SLR) Frequencies and Resolution onto Step Detection Sensitivity

As summarized in Table 5, the SLR-10 has a significantly higher sensitivity (in terms of correctly detected steps) in comparison to the slower SLR-04. Since these variations in sensitivity might directly cover a specific type of walks, the systems’ overall sensitivity to detect critical changes in gait might thereby exclude specific relevant walks (e.g., the slower one). In our perspective this is a relevant limitation that contradicts the applicability off the SLR-04 for this purpose. These results agree with the lower sensing quality of the SLR-04 and can be seen as a result from the lower measurement characteristics (namely the max. measurement distance, frequency and resolution). Thus, we further only considered the SLR-10 and excluded the SLR-04 from subsequent evaluations.

### 3.3. Sensitivity of Gait Parameters

For the subsequent separated grouped analysis for the walks with normal walking pace and the ones with fast walking pace, 254 walks could not be considered, since they were unrelated to the blocks of normal and fast walking, even though representing generally valid measures. This exclusion would not be required for normal use of the UGMO, but was only required for the subsequent evaluation.

Among the correctly detected 7623 walks, a further 144 walks (1.9%) had to be removed due to the following reasons:

- 75 walks (73 GAITRite and 2 SLR; 59 of them occurring in the fast pace group) had a distance of less than 3 m and thus were removed automatically (even though shorter distances can be expected to achieve reliable results either, we defined it as a min requirement to achieve comparability among the 4.88 m active GAITRite and the SLR).
- For 60 walks (0.8%; among them 48 of them occurred in the fast pace group) no gait parameters could be calculated due to an error in the SLR data representation.
- 9 walks (6 SLR and 3 GAITRite; all in the fast pace group) included stride-length of more than 2 m and thus were removed automatically

These filter steps are integrated in the UGMO platform, to exclude medically meaningless/erroneous measures.

With the remaining correctly detected 7479 walks and 55,690 steps for UGMO and 48,011 for the GaitRite on a distance of approximately 34.8 km over approximately 7 h, UGMO’s sensitivity to the gait parameters shown in Table 7 was evaluated in comparison to the GAITRite as reference. In order to classify UGMO’s sensitivity regarding varying walking speeds the investigated walks were separated regarding the walking speeds into normal pace (3345 considered walks) and fast walking pace (4134 considered walks).

## 4. Discussion

Due to the study’s population size and the wide distribution of subjects ages—ranging from 21 to 82 years with a mean age of 59.6 years and a standard deviation of 22.8 years—the results can be expected to be representative for the intended purpose to detect functional decline in aging adults.

In regard to the influence of the SLR type (as associated to varying data rates, measuring areas and angular resolutions), the SLR-10 achieves a much higher sensitivity in terms of the correct detection of steps (98% compared to 77%) and walks (97% compared to 66%) than the cheaper SLR-04, whose lower performance might have been affected by the sensors range, frequency and angular resolution characteristics. Thus, the SLR-10 should be applied instead of the SLR-04 to ensure the correct detection of most walks. With the rate of correct positive detected walks by the SLR-10—as compared to the GAITRite acting as reference measure—the UGMO is well suited to act as monitoring device. Thus, we could confirm UGMO’s high sensitivity to detect bypassing walks automatically.

**Figure 9:**
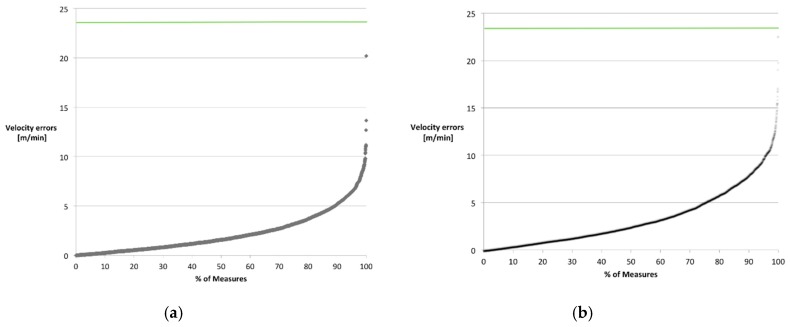
Error distribution of velocity measures separated by groups for (**a**) normal walking pace and (**b**) fast walking pace; green lines (at 23.8 m/min) identifies the minimal expected variance for common age- and disease-related variations.

Considering UGMO’s (with SLR-10) sensitivity regarding the common gait parameters velocity and stride length, the results are similarly promising. With UGMO’s corresponding sensitivity being sufficient to detect typical age- and disease-related variations for velocity (as summarized in Table 8), UMGO’s applicability to detect these meaningful variations for functional decline, since its 99 percentile error is well below the critical measurement range. Furthermore, the Pearson correlation coefficient is with 0.95 and 0.93 excellent for both considered walking paces [43]. As shown in Figure 9 all errors reside below the age- and disease-related variations and 95% and 81% of the measurement for normal and fast walking pace groups hold errors below a fourth of the minimal age- and disease-related variations. Since the remaining measures with higher errors were well distributed among the subjects, UGMO’s velocity is sufficiently sensitive, especially if applied for repeated measures—an approach that is practical for both supervised and unsupervised settings.

Similarly, the stride length correlated with 0.91 to 0.96 excellent [43] and all errors at 99 percentile are well within the margin of age- and disease-related variations in stride length for both speeds and legs (compare Table 8). Thus, the sensitivity of UGMO’s stride-length calculation could be confirmed as well.

**Figure 10:**
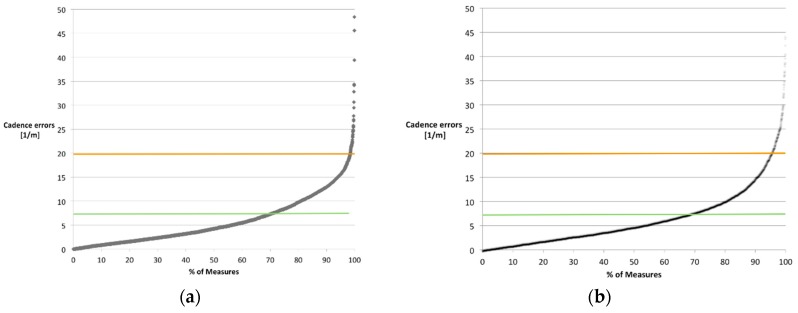
Error distribution of cadence measures separated by groups for (**a**) normal walking pace and (**b**) fast walking pace; green and orange lines (at 7.3 and 19.6. steps/min) identifies the minimal expected variance for common age- and disease-related variations and maximal ones respectively.

The sensitivity of UGMO’s cadence measure is rather ambiguous (see Figure 10). With a correlation of 0.71 and 0.79 for the normal and fast walking paces, its sensitivity is rather modest [43]. Furthermore, only 71% and 87% of the measures in the normal and respectively fast walking pace groups fall below the minimal age- and disease-related variations. Consequently, the cadence parameter might only be sensitive if applied via repeated measures.

Comparing the UGMO’s sensitivity to the reported ones of both existing systems, the velocity and stride-length are representative of existing SLRs (as summarized in Table 3) and corresponding depth-sensor based systems as summarized in [44]. For the velocity, the error is comparable to the reported errors of 3.84 m/min of other SLR systems. The achieved correlation of 0.95 to 0.93 is slightly below those of Kinect-sensor based systems, which regularly achieve correlations of up to 0.99. With the correlation of 0.95 and 0.93 UGMO’s stride length is with median errors around 2.3 to 3.4 cm on par with the reported mean and root mean square errors of approximately 3 cm for other SLRs (see Table 3), despite the different viewing angles. The correlations of the stride lengths are slightly below Kinect-based systems, which correlated up to 0.99 compared to UGMO’s 0.91 to 0.96. However, most of the Kinect-based systems have been evaluated for use with a frontal view on the movement.

The stance- and swing-time parameters correlate modestly with the once of the GAITRite. This might be related to the variation in the measurement setup—due to the UGMO’s extended sensing area compared to the GAITRite. This might result in only UGMO detecting the potential variations in swing-time at beginning and ending of the walkway as transitioning to/from turning.

In general, the results indicate that some outliers occurred among all considered parameters (consider the large margin between the 99 and 100 percentile in Table 7), which intentionally have not been trimmed. These outliers might be related to the additional steps that have been recorded only by the UGMO and not by the GAITRite (as a consequence of UGMO’s wider viewing angle, see walk distance in Table 7) and might include suddenly altered movements during transitioning to turning and, thereby, might rarely occur in realistic settings.

Furthermore, such rare outliers can be even filtered within UGMO by considering only consistent variations over multiple subsequent walks as relevant indicators for functional decline.

Since the variations of UGMO’s sensitivity among the gait parameters for the normal and fast walking speed groups (see Table 7) were rather low, the variations of the sensitivity for slower walking speeds could be expected to be comparable as well, especially since the SLR will have more samples per step to be considered in the calculations. For velocity, the error decreases and the correlation becomes stronger with lower walking speeds, while for stride length the correlation decreases slightly with decreasing walking speeds.

Additional validations of the UGMO system are intended to confirm the system’s sensitivity for slower walking speeds, different viewing angles, minimal and maximal distances and non-rectilinear walks among other influences expected for unsupervised applications. The current system is also not able to detect walks covering subjects in-between stop and or turn around, during which pets walk through the measuring area, or other conditions that might occur in a real-world setting. Thus, further developments in that regard are intended.

## 5. Conclusions

The article introduces the hardware and signal-processing tool chain of the unobtrusive gait monitoring (UGMO) system that detects variations in common gait parameters (such as velocity and stride length) of elderly people that have been shown to be a reliable indicator for functional and cognitive decline. Functional and cognitive decline has been shown to be strongly correlated to fall risks and being a leading cause of fatal injury and the most common cause of non-fatal trauma-related hospital admissions among older adults causing over $50 billion total medical costs in 2015. The novelty of UGMO in comparison to other SLR-based systems is that it supports the lateral supervision of walks (in contrast to the commonly used frontal recording), which is especially challenging due to the hidden leg problem, to be considered. Thereby, UGMO is expected to be highly applicable for unsupervised home assessment. By evaluating the UGMO system in comparison to the GAITRite, as a reference system, via 86 study participant with ages ranging from 21 to 82 years, with a mean age of 59.6 years and a standard deviation of 22.8 years, covering each passing twice the 6 min walk test under supervision, the following findings have been gained: comparing two SLR types, it has been shown, that the SLR-10 achieves a much higher sensitivity than the cheaper SLR-04 in terms of the correct detection of steps (98% compared to 77%) and walks (97% compared to 66%).

By using the SLR-10, the UGMO sensitivity in measuring gait parameters such as gait velocity and stride length is sufficient to detect reported age- and disease-related variations. Consequently, UGMO has been shown in a standardised supervised setting to be suitable to detect functional decline as associated with an increased fall risk. With the UGMO’s applicability being confirmed within standardised settings (requiring lateral and continuous walks) we are looking forward to enhancing the system for more general scenarios and confirm its applicability to unsupervised unstandardised settings via additional studies.

Aside from their use for continuous monitoring within predestined groups, these sensors can also be expected to enhance knowledge about the reasons for falls and the impact of environmental factors’ such as lighting conditions, daytime, flooring materials, obstacles, tiredness and activity levels on the quality and stability of gait. The associated causes and conditions of falling as a consequence of unstable gaits, being a fundamental requirement to implement safer environments, are not yet fully understood. Consequently, in order to gain insights on the genuine characteristics of gait and the related influences of environmental factors (such as lighting conditions or flooring), the UGMO represents a suitable sensor to generate new insights in this regard.

## Figures and Tables

**Table 1:** Common gait parameters and corresponding algorithms.

Gait Parameter	Description	Algorithm	Separately per Foot
Step length [m]	Distance between the heel of one foot to the heel of the next one along walking direction (see left and right step in Figure 1)	Distance passed from toe off to initial contact.	x
Cadence [1/min]	Step frequency	Number of steps/time [min]	
Walking velocity [m/min]	Gait speed	Distance [m]/time [min] = stride_length [m] × cadence [1/min]/2	
Stance time [s]	Time each foot has contact with the ground per step (see left and right stance in Figure 1.)	Last_contact_time – first_contact_time	x
Swing time [s]	Time each foot has no contact with the ground per step (see left and right swing in Figure 1)	Step[i + 1]. first_contact_time – step[i]. last_contact_time	x
Stride length [m]	Distance from one foot hitting the ground to its next ground contact (see full gait cycle shown in Figure 1 as example for right stride).	Step_length_Left_ + step_length_Right_	x

**Table 2:** Measures for common gait parameters as an indication of clinical meaningfulness ranges as mean (^+^ The selected columns as well specify standard deviation as ±SD and mean variation from the normal gender independent value in % in parenthesis).

Parameter	Men Normal [20]	Women Normal [20]	Normal (Gender Independent) [21]	Transition to Frailty^+^ [16]	Fearful Fallers ^+^ [22]
Velocity [m/min]	86 (80–91)	77 (73–81)	82	58.2 ± 13.8 (29%)	39.6 ± 11.4 (51%)
Cadence [1/min]	111	117	113	105.7 ± 12.7 (6%)	95.4 ± 11.2 (15%)
Stride length [m]	1.46	1.28	1.41	1.11 ± 0.18 (21%)	0.83 ± 0.16 (41%)

**Table 3:** Existing ambient gait detection systems, their considered parameters, sensitivities, and study population and orientation (F: frontal, B: backwards, S: lateral, n.d.: indicating no information, BGS: background subtraction, DR: Doppler radar, EDF: erosion-dilation filter, GNN: global nearest neighbor, HMM: hidden Markov model, IQR: interquartile range; KC: 3D motion recorder™Kissei Comtec, KF: Kalman filter, KM: K-means, LMS: least mean square, PF: particle filter, RMSE: root mean square error, SD: standard deviation, Scanning laser rangefinders (SLRs): 04-LX-UG01: SLR URG04-LX-UG01, 04LX-F01: SLR UBG-04LX-F01, 30LX: SLR UTM-30LX.

	Sensor System			Resulting Sensitivity				Study Design	
Year	Sensor	Algorithm	Orient/Height	Detection Rate of Steps/Walks (in %)	Gait Velocity/Speed Error (cm/s)	Stride Length Error (cm)	Swing Time Error (s)	Reference System	Subjects (m/f); Age
2015 [28]	04-LX-UG01, UTM-30LX	KF, GNN	F 27 cm	Leg tracking (97.1%) Foot contact (99.3%)	n.d.	3.5 cm	n.d.	Vicon	7 (4/3); 70.9 years ± 3.5
2017 [29]	04LX-F01	KF/PF	F 40 cm	KF: 18.4% (10.5–30.1%) PF: 0.6% (0–2.3%)	KF: 7.9 (4.5–11.3) cm/s PF: 6.4 cm/s (4.2–9.9) RMSE	n.d.	n.d.	Vicon	4; 65 + years
2016 [30]	04LX-F01	HMM, KM, KF	F 40 cm walker	n.d.	n.d.	HMM: 8.9% ± 5.2% RB: 10.1% ± 6.8%	HMM: 10.9% ± 6.2% RB: 16.9% ± 5.7%	GAITRite	5; 65 + years
2014 [31,32]	SLR, 4 force sensors	BGS, DIET [13]	F/B 35 cm	n.d.	n.d.	3 cm	0.08 s	SIMI Motion	7 (5/2); 23–31 years
2009 [33]	UTM-30LX	LMS fitting [34]	F 10 cm	n.d.	n.d.	1.1 SD 0.8 cm	n.d.	n.d.	6 (3/3); n.d.
2017 [35]	UTM-30LX	n.d.	F 25 cm	n.d.	n.d.	25.9 cm (3.37%) ± 23.8 cm (3.53%)	0.091 s (22.9%) ± 0.051 s (12.1%)	KC	34 (21/13); 22–30 years
2015 [36]	UTM-30LX	KF, Catmull–Rom spline [37]	F/B	96.4% (tracking success rate)	n.d.	3–5 cm RMSE	n.d.	Vicon	7 (6/1); 23.0 ± 1.9 years
2017 [38]	Kinect	BGS [39] LSM, EDF, HMM	F	n.d.	0.3; SD 0.12 cm/s	0.6; SD 8.31 cm	n.d.	GAITRite	11 (7/4); 22–53 years
2017 [40]	DR, Precision Line RCR-50	Zero-crossing in time domain IQR filter	F 15 cm	n.d.	1.08 RMSE	n.d.	n.d.	Vicon, Kinect	8; seniors

**Table 4:** Characteristics of the considered SLR.

SLR Scanner-Type	Hokuyo URG-04LX (SLR-04) [41]	Hokuyo UST-10LX (SLR-10) [42]
**Scanning rate**	10 Hz	40 Hz
**Measuring area**	2–560 cm	6–1000 cm
	240°	270°
**Measuring Accuracy**	±10 mm (0.20–1 m), ±3% (1–5.6 m)	±40 mm
**Angular resolution**	approx. 0.36°	approx. 0.25°
**I/O**	Universal series bus (USB)	Local-area network (LAN)
**Power Supply**	5V DC, 500 mA (800 mA during start up)	12VDC/24VDC, 150 mA (450 mA during start up)

**Table 5:** Suitability of synchronization overlap (bold indicating the selected optimum).

Synchronisation Overlap (s) between SLR and GAITRite	Number of Steps (% of Overall 56351 Considered Steps)	
	URG04LX	UST-10LX
4	43162 (75.6%)	55083 (97.7%)
3.5	43253 (76.8%)	55167 (97.9%)
**3**	**43260 (76.8%)**	**55181 (97.9%)**
2.5	43092 (76.5%)	55188 (97.9%)
2	42035 (74.6%)	55153 (97.9%)
1.5	37240 (66.1%)	54516 (96.7%)
1	25221 (44.8%)	46879 (83.2%)
0.5	11473 (20.4%)	23961 (42.5%)

**Table 6:** The descriptive statistics of the considered cohort of 86 subjects (including 39 females). The leg-length was measured from the top of trochanter major till the bottom of the ankle and thus, represents the length of upper and lower limbs.

	Average	SD	Min	Max
**Age (years)**	59.6	22.8	21	82
**Height (cm)**	173.3	10.2	151	196
**Weight (kg)**	76.1	12.8	53	103
**Left leg-length (cm)**	80.8	5.59	67	94
**Right leg-length (cm)**	80.79	5.65	67	94

**Table 7:** UGMO’s gait analysis sensitivity in comparison to GAITRite. The table shows the interquartile ranges and the 99 percentile over the median errors (SLR-GAITRite) over all walks per subject as error = abs(SLR value) = abs(GAITRite value) between SLR-10 and GAITRite; CC: Pearson correlation coefficient.

Parameter	Walking Speed	Min	1. IQR	Median	3. IQR	99%	Max	CC
**Velocity [m/min]**	**normal**	0	0.68	1.58	3.17	8.8	20.19	0.95
	**fast**	0	1.08	2.46	5.03	12.96	45.81	0.93
**Left Stride Length Mean (cm)**	**normal**	−68.66	−5.43	−2.49	−1.50	1.85	18.03	0.91
	**fast**	−53.59	−3.92	−2.33	−1.41	2.22	16.45	0.95
**Right Stride Length Mean (cm)**	**normal**	−56.46	−5.56	−3.30	−2.15	1.24	17.76	0.93
	**fast**	−42.75	−5.17	−3.44	−2.22	0.93	7.51	0.96
**Cadence [1/min]**	**normal**	−19.6	0.1	3.2	8.4	28.66	111.4	0.71
	**fast**	−23.4	−1.8	2.6	6.8	28.94	66.3	0.79
**Left Stance Time Mean (s)**	**normal**	−0.56	0	0.03	0.05	0.12	0.23	0.75
	**fast**	−0.47	−0.02	0	0.03	0.09	0.54	0.81
**Right Stance Time Mean (s)**	**normal**	−0.53	−0.10	−0.05	0	0.09	0.14	0.66
	**fast**	−0.43	−0.11	−0.058	−0.01	0.07	0.12	0.61
**Left Swing Time Mean (s)**	**normal**	−0.28	−0.04	−0.02	0	0.14	0.41	0.49
	**fast**	−0.17	−0.01	0.01	0.02	0.19	0.45	0.56
**Right Swing Time Mean (s)**	**normal**	−0.18	−0.04	−0.02	0	0.15	0.41	0.51
	**fast**	−0.23	−0.01	0.01	0.02	0.13	0.40	0.65
**Walks’ Distance [m]**	**normal**	0	0.43	0.63	1.03	1,64	1.95	n.a.
	**fast**	0	0.39	0.63	1.04	1.58	2.08	n.a.

**Table 8:** The sensitivity of UGMO’s gait parameters with the SLR-10 in comparison to the expected variations as associated with common age- and disease-related variations (calculated as the difference between the normal gender-independent value and the corresponding disease-specific value, as discussed in Table 2).

	Common Age- and Disease-Related Variations		Error of SLR as 99 Percentile (Normal/Fast Walking Pace)
Parameter	Min [16]	Max [22]	
**Velocity Diff [m/min]**	23.8	42.4	8.8/12.96
**Stride Length [cm]**	30	58	1.85/2.22
**Cadence Diff [1/min]**	7.3	19.6	28.66/28.94
